# Exploring the different cognitive, emotional and imaginative experiences of autistic and non-autistic adult readers when contemplating serious literature as compared to non-fiction

**DOI:** 10.3389/fpsyg.2023.1001268

**Published:** 2023-05-04

**Authors:** Melissa Chapple, Philip Davis, Josie Billington, Rhiannon Corcoran

**Affiliations:** ^1^Department of Primary Care and Mental Health, School of Psychology, University of Liverpool, Liverpool, United Kingdom; ^2^Centre for Research Into Reading, Literature and Society, University of Liverpool, Liverpool, United Kingdom

**Keywords:** autism, empathy, literary fiction, non-fiction, neurodiversity

## Abstract

**Introduction:**

Recent research has demonstrated how reflections on serious literature can challenge dominant social-deficit views of autism. This method enables autistic readers to explore social realities more slowly and carefully, encouraging detail-focused considerations. Previous research has also shown that autistic and non-autistic readers reflecting on serious literature together are able to achieve mutuality in a way that enables them to overcome the double empathy problem. However, the advantages of reading aloud designs have yet to be explored with autistic and non-autistic readers due to previous concerns amongst autistic people on the issue of being read aloud to. The present study aimed to explore how an adapted shared reading design that compared serious literature and non-fiction would enable autistic and non-autistic readers to imaginatively engage in the reading experience.

**Methods:**

Seven autistic and six non-autistic participants read 8 short text extracts alone while listening to pre-recorded audio of an experienced reader reading each text aloud. Participants completed a reflective questionnaire for each text and a follow-up interview where moving parts of the text were then re-read aloud before discussion. Half of these texts were serious literature, while the other half were non-fiction. Similarly, half of the texts explored fictional social realities that depicted a lack of mutuality, or non-fiction accounts of autism; while the other half explored broader emotional experiences.

**Results:**

Thematic and literary analysis of participant reflections and follow-up interviews revealed three main themes: (1) From Surface Reading to Intuitive Engagement, (2) Imaginative Feeling and (3) Going Forward from the Reading Experience.

**Discussion:**

The findings showed that autistic readers were better able to hold onto the detailed complexity of serious literature, while non-autistic readers tended to reduce information down to key ideas and understandings for later generalization. Findings are discussed in relation to future shared reading designs.

## 1. Introduction

Autism broadly refers to developmental differences that influence how a person might think, feel and interact with the world around them (Fletcher-Watson and Happé, 2019). However, beyond these broad categories of difference, it is hard to refine the definition of autism in a way that does not over-simplify the complex experiences of autistic^1^ people (Fletcher-Watson and Happé, 2019; Botha, 2021). While there have been many attempts to understand common socio-cognitive processing differences amongst autistic people, one key hurdle is the over-dominance of the medical model of autism (Waltz, 2013). This model positions autism as a deficiency of human development, treating human difference in the same way as physiological disease (Kinderman et al., 2013; Waltz, 2013). Current diagnostic definitions of autism center upon assumed key deficits in social communication, repetitive behavior and restricted interests (Murray et al., 2005; American Psychiatric Association, 2013). What has resulted is a dominant narrative of disorder that has further led to harmful pursuits toward the prevention and cure of autism (Waltz, 2013; Milton et al., 2020). In wider society, pathological, deficit-focused views of autistic people have resulted in stigma and subsequent discrimination (Green et al., 2005; Pearson and Rose, 2021).

In particular, theoretical models of autism have often been underpinned by deficit views, in a way that subsequently reinforces pathologized understandings of autistic people (for example: Baron-Cohen, 1997, 2002, 2009; Happé, 1999). Specifically, the weak central coherence (WCC) theory (Happé, 1999) argues that autistic people attend more to detail, at the expense of integrating information into broader contexts (Happé, 1999; Hill, 2004). Within social situations, this would mean resultant difficulties in understanding overall interactions. Specifically, the WCC would assume difficulties in generalizing social learning across situations, which may be linked to a tendency for feeling socially overwhelmed amongst autistic people (Happé, 1999; Hill, 2004). While the theory has been criticized for failing to specify the level at which integration difficulties may occur (Baron-Cohen, 2008), the idea that autistic people attend more to detail has remained influential (Murray et al., 2005; Lesser and Murray, 2020). The theory of monotropism (Murray et al., 2005) furthers the idea that autistic people have a tendency to attend to detail. This theory positions autistic people as being able to integrate information into wider contexts, but it does still suggest that autistic people might find it more difficult to process multiple streams of information (Murray, 2020). Therefore, both monotropism and WCC position autistic people as struggling with social breadth, or the ability to model other minds (Happé, 1999; Lesser and Murray, 2020). The theories then suggest that typically developing people would tend to better understand social breadth at the expense of depth (Lesser and Murray, 2020).

Similar claims have been made by the mindblindness theory of autism (Baron-Cohen, 1997), which argues that autistic people struggle to imaginatively represent the minds of others, known as theory of mind. The theory argues that autistic people are extremely egocentric, applying their own mental states to others regardless of similarity to self or context (Lombardo and Baron-Cohen, 2011; Bodner et al., 2015). Despite the pervasive influence of this theory, findings have contradicted these assumptions. Specifically, autistic people have instead been found to view themselves through an imagined third-person perspective (Burrows et al., 2017; Arnaud, 2022). This contrasts with a general bias for prioritizing first-person self-assessments that is often observed within non-autistic, Western samples (Burrows et al., 2017; Arnaud, 2022). The reason for this difference appears to result from a sense that autistic people are less likely to trust their own perspectives for self-evaluations, feeling instead that others know them better than they do themselves (Schriber et al., 2014). These findings counter the mindblindness theory by showing a complex mobility of perspective while also raising concerns around whether deficit-based views of autism lead to reduced confidence in self and ability amongst autistic people. Early versions of the empathizing-systemizing (E-S) theory furthered these deficit views by claiming that autistic people are broadly less empathic than their non-autistic peers (Baron-Cohen, 2002, 2009). Instead, autistic people are argued to possess a processing style that is more systematic in nature (Baron-Cohen, 2002, 2008, 2009). Here, systemizing refers to the ability to extract regularities when observing a process in order to establish rules that govern it and make predictions about future events and consequences (Baron-Cohen, 2008). This approach to understanding socio-emotional information is then seen as too rigid and mechanical to successfully infer and predict the feelings and behaviors of others (Baron-Cohen, 2008).

As a result of these empathic deficit views, there has been a long-standing research focus examining the ways in which autistic people might differently empathize with others (Dinishak and Akhtar, 2013; Hume and Burgess, 2021). However, the term empathy, much like the term autism, can be difficult to define in a way that does not narrowly reduce the concept down into too-easily understood, restrictive criteria that fail to capture the complexity of feelings being referenced (Fletcher-Watson and Bird, 2020). Broadly, the term is often taken to refer to the inter-related abilities to recognize, predict, feel through, and respond to the feelings of others (Harmsen, 2019; Fletcher-Watson and Bird, 2020). Research on autistic experiences of empathy has generally concluded that autistic people struggle to take the perspective of others (Smith, 2009; Song et al., 2019) and recognize the emotions of others (Gaigg, 2012; Rigby et al., 2018). However, research is often based on cognitive tests that favor fast-paced and conclusive assumptions made on the basis of limited sets of information (Fletcher-Watson and Bird, 2020). Findings then lack ecological validity as a result of the research failing to mirror everyday socio-emotional experiences which often allow for and benefit from more careful, complex considerations (Fletcher-Watson and Bird, 2020). These slower and more careful empathic assessments may be more common amongst autistic people (Chapple et al., 2022), putting them at a disadvantage when tested with the standardized cognitive tests available (Fletcher-Watson and Bird, 2020).

Furthermore, social deficit accounts of autism fail to account for the bi-directional nature of social interactions (Milton et al., 2018). Milton’s (2012) double empathy problem highlights a need to understand that mutuality and context are developed within a given interaction. Therefore, social skills are not something to be objectively learnt and generalized as they are so often described (Milton, 2012). Rather, the difficulties often observed when autistic and non-autistic people interact, as Milton (2012) calls problems of double empathy, are positioned as stemming from mutual difficulties in understanding one another’s perspective, which has been observed across research (Milton, 2012; Edey et al., 2016; Sheppard et al., 2016; Heasman and Gillespie, 2019; Crompton et al., 2020b). The differing experiences, norms and methods of communication between autistic and non-autistic people make this failure to find mutuality more likely than when each interacts with someone who shares their neurotype (Milton, 2012; Morrison et al., 2020). For the typically developing population, these mixed-neurotype encounters are rare due to the much greater likelihood of them encountering people who share their neurotype in everyday life (Milton, 2012; Chown, 2014). The result is that autistic individuals are then typically blamed by non-autistic people for socio-communicative difficulties resulting from the struggle to build mutuality and achieve reciprocity (Milton, 2012; Chown, 2014). Conversely, autistic people are more likely to have to navigate a lack of mutuality in their daily lives as a result of belonging to a neurominority (Milton, 2012; Chown, 2014; Botha, 2021). As a result, autistic people may be less likely to assume pre-set norms, taking more time to identify common ground and to develop shared social understandings (Milton, 2012; Chown, 2014; DeBrabander et al., 2019; Chapple et al., 2021a,^2022^). Research has supported this, showing that autistic people interacting together can achieve mutuality (Milton, 2012; Heasman and Gillespie, 2018; Crompton et al., 2020a,c; Morrison et al., 2020) even after initial negative impressions (DeBrabander et al., 2019).

To move understandings of autistic people away from deficit-focused views, research methods that involve more open, empathic thinking about autistic people are needed (Ida, 2020; Chapple et al., 2021a). One ecologically valid method that can offer this type of exploration is the contemplation of fiction (Chapple et al., 2021a,^2022^). This is because fiction provides social simulations that mirror the real social world, making the experience feel like a live reality (Mar and Oatley, 2008; Mumper and Gerrig, 2019). Specifically, fiction encourages complex movements between a reader’s own perspective, character perspectives and the inferred perspective of the author (Mar and Oatley, 2008; Zunshine, 2011; Waytz et al., 2015). This perspective mobility activates past, personal memories that enable readers to respond empathically with the minds in the text (Mumper and Gerrig, 2019). Rather than these assimilations encouraging readers to egocentrically impose their own perspective, moving parts of a text become part of the reader, allowing them to feel together with the minds held by the text (Zunshine, 2011; Limburg, 2021). In this way, the fiction is able to hold empathy for its readers, making the shared feeling a complex two-way sharing (Limburg, 2021). Serious literature is thought to be particularly evocative of these experiences, encouraging readers to mentally “do” the literature rather than passively read it (Barnes, 2018; Davis, 2020). Serious literature is here used to refer to fiction that engages readers with significant human situations through the use of powerful, moving language (Davis, 2020; Davis and Magee, 2020). This powerful language encourages readers to hold onto feelings of being moved (O’Sullivan et al., 2015; Davis, 2020). The result is that readers explore the uncertainties and complexities of imagined social realities more carefully, holding onto ambiguity in a way that makes room for deeper empathic feelings (O’Sullivan et al., 2015; Chapple et al., 2022). Although serious literature does not necessarily refer to classic texts, older literature can be particularly powerful due to its ability to “regenerate” modern contexts through representations of core human feelings that transcend time (Farrington et al., 2019).

Through this movement, the reading experience prevents overly conclusive judgments that are implemented when generalizing from learnt social scripts (Djikic et al., 2013; O’Sullivan et al., 2015). Instead, serious literature encourages readers to find value in the intangible, staying with moments of movement from intangible feelings before turning them into something more easily recognizable (Farrington et al., 2019). Therefore, serious literature creates social realities for readers that are arguably more emotionally complex than everyday experiences (Farrington et al., 2019). This is because reading can help readers overcome satiation with default, normative ways of thinking that can prevent us from holding onto and feeling with emergent live thoughts (Farrington et al., 2019; Davis and Magee, 2020). Shared reading in particular can bring readers from different walks of life together in ways that encourage an overcoming of any pre-conceived prejudice toward different minds (Longden et al., 2015; Chapple et al., 2021a). Where readers are moved to feel with one another through shared thinking together, openness and empathic feeling are supported (Longden et al., 2015; Chapple et al., 2021a). Specifically, reading allows social risk taking, where readers can begin to feel and think with different Others, regardless of any perceived personal or social risks from mutual identification and feeling (Koopman and Hakemulder, 2015). This social risk taking can occur by thinking and feeling with Othered minds within a text (Koopman and Hakemulder, 2015) or with Othered readers through shared reading reflections (Farrington et al., 2019). Longden et al. (2015) report that the liveness of being read aloud to in a shared reading group is particularly important in surprising readers out of default thinking and into holding in mind live thoughts and feelings.

Texts that engage readers with human adversity are thought to be particularly moving (Strick and Van Soolingen, 2018; Davis, 2020). Importantly, research has demonstrated that when autistic and non-autistic people reflected on a text addressing human adversity, there resulted an overcoming of stigma and the double empathy problem (Chapple et al., 2021a). Current findings indicate that while reading alone, autistic people hold onto complexity, meaning they read in more literary ways that enable them to benefit from both the emotional depth and social breadth of literature (Chapple et al., 2022). However, earlier findings that autistic people might feel uncomfortable with the idea of reading together with others or being read to (Chapple et al., 2021b) mean that explorations have so far been designed around autistic people reading alone (Chapple et al., 2021a,^2022^). Therefore, the previously demonstrated value of live reading (Longden et al., 2015) has yet to be applied to shared reading between autistic and non-autistic readers. However, it is first important to explore how autistic people engage with and benefit from reading aloud designs in more comfortable settings, such as while being able to read alone.

Considerations of text type should also be given for autistic readers (Chapple et al., 2021b). Specifically, autistic adults have highlighted a need for social experiences within texts to be relatable in order to achieve immersed feeling (Chapple et al., 2021b). Similarly, it has been suggested that autistic people may prefer non-fiction (Baron-Cohen, 2008; Barnes, 2012). Although research has demonstrated that autistic people do enjoy and engage with fiction (Barnes, 2012; Davidson and Ellis Weismer, 2018; Armstrong et al., 2019; Chapple et al., 2021b), qualitative research has highlighted that autistic people can find emotional value in reading biographical non-fiction and factual non-fiction that relates to specialized interests (Chapple et al., 2021b). Arguably, serious literature contains autobiographical elements within it, due to the author’s own personal involvement in the fictional narrative (Zunshine, 2011; McCartney, 2021). However, it is important to explore how autistic and non-autistic readers would engage with more informal autobiographical works in order to explore how these accounts would compare to fictional representations of human difference and adversity.

The current study aimed to address these considerations by exploring how autistic and non-autistic readers would engage with various text types through a distanced reading-aloud design. Specifically, the study aimed to answer two questions: (1) how do autistic adult readers engage with serious literature compared to non-fiction and how does this compare to non-autistic adult readers? And (2) could texts depicting the double empathy problem or autistic experiences provide benefits for autistic and/or non-autistic readers compared to texts exploring broader human experiences? To explore these questions, participants read 8 short text extracts alone while listening to pre-recorded audio files of an experienced reader^2^ reading each text aloud. The texts were varied by whether they represented autistic experiences or broader human experiences and also by genre (Section “2.3. Study materials”).

## 2. Materials and methods

### 2.1. Participants

Initially, participants were invited from a database of individuals who had previously been involved in reading research at the University of Liverpool and had given their consent to be contacted about future research. Further participants were then recruited through social media and local advertisements. Initially, 40 individuals participated in the screening process, 15 of which were not enrolled into the wider study due to not meeting the inclusion criteria. A total of 25 participants were invited to take part in the study, with 12 dropping out of the study without reason, resulting in the removal of their data. Participants were invited into the study until the research team agreed that data saturation had been reached within each group (autistic, non-autistic). Inclusion criteria included being 18 or over, having proficient English language skills and scoring an estimated Wechsler Adult Intelligence Scale (WAIS) IQ score of 90 or above as assessed by the quick test (QT) (Ammons and Ammons, 1962). For autistic adults who did not have an official diagnosis (i.e., who self-identified as autistic), there was an exclusion criterion of scoring below 32 (the suggested cut off for autism) on the autism quotient (AQ) (Baron-Cohen et al., 2001). Undiagnosed autistic participants were included to take account of accurate gender representation due to the longstanding underdiagnosis of women and genders outside binary norms (Fletcher-Watson and Happé, 2019). Non-autistic participants had an additional exclusion criterion of scoring over 32 on the AQ.

Overall, thirteen participants took part in this research study (see Table 1 for demographics). Seven were autistic (male *N* = 3; female *N* = 2; gender outside binary norms *N* = 2) aged 22–48 (*M* = 34.57, SD = 9.31) and six were non-autistic (male *N* = 3, female *N* = 3) aged 24–34 (*M* = 28.33, SD = 4.23). All participants were invited to take part in a follow-up interview about their text responses, with only one participant (autistic) choosing not to take part. Six (4 autistic) participants had previously taken part in reading research led by the team. This study was approved by the University of Liverpool Research Ethics Committee.

**TABLE 1:** Participant demographics.

Participant no.	Age	Gender	AQ	IQ (WAIS equivalent)	Level of education completed	Neurotype
4	41–50	Female	38	116	Doctoral training	Autistic: diagnosed
7	31–40	Gender non-conforming	36	102	PGCE	Autistic: diagnosed
8	31–40	Female	34	116	Doctoral training	Autistic: ongoing assessment
10	21–30	Gender non-conforming	43	108	Bachelors	Autistic: diagnosed
11	21–30	Male	40	96	GCSE	Autistic: diagnosed
12	41–50	Male	45	98	A level	Autistic: diagnosed
19	21–30	Male	48	135	Masters	Autistic: diagnosed
25	21–30	Male	9	104	Foundation degree/diploma	Non-autistic
26	31–40	Female	22	104	Doctoral training	Non-autistic
28	31–40	Female	7	104	Masters	Non-autistic
30	21–30	Female	15	100	Masters	Non-autistic
38	21–30	Male	6	110	Bachelors	Non-autistic
40	31–40	Male	6	120	Foundation degree/diploma	Non-autistic

### 2.2. Screening measures

A demographics questionnaire asked for participants’ age, gender and highest completed qualification. Eligibility questions were also asked at this stage.

#### 2.2.1. The autism quotient (AQ)

The AQ (Baron-Cohen et al., 2001) is a 50-item questionnaire that uses statements to elicit a score designed to reflect autistic traits in clinical and non-clinical samples. The AQ was used to assess the number of self-reported autistic traits in both samples.

#### 2.2.2. The quick test (QT)

A single 50-item version of the QT (Ammons and Ammons, 1962) was used to assess participants’ comprehension abilities. The test involves participants looking at 4 pictures and deciding which picture each word goes best with. Given the age of the QT, the raw test score is converted to a WAIS, not WAIS-R, equivalent IQ. This was considered an adequate method for obtaining a rough estimate of reading comprehension ability for this study where its brevity was an asset and where IQ data was not going to be subjected to further analysis.

### 2.3. Study materials

Participants read 8 three-page long text extracts which were split into two groups: (A) texts exploring human disadvantage in a way that was judged by the research team as demonstrating the double empathy problem (Milton, 2012) or autistic experiences and (B) texts exploring wider human disadvantage and related emotion in everyday situations. The texts in group A were judged as representative by the first author, who is autistic, and by an autistic research assistant who left the project due to time constraints. All texts were chosen with guidance from the 2nd and 3rd authors, who are experienced English literary scholars and come from The Reader Organization’s recommended texts for shared reading (Macmillan, 2010). Extracts that depicted abuse were avoided due to fear of triggering memories of abuse and post-traumatic stress disorder (PTSD) which has higher prevalence amongst autistic people (Rumball et al., 2021). Although the included text *Eleanor Oliphant is Completely Fine* (Honeyman, 2017) explores themes of inter-personal trauma, the short extract from this text that was used within this study did not contain any instances of or references to trauma. Additionally, all final extracts stated the text from which the extract was taken and gave a brief background to the text to create immersion and alert readers to anything that they may not want to read for personal reasons. Within each of the two groups, there were 4 types of text: (1) classic literature, (2) contemporary literature (2010–2020), (3) scientific non-fiction and (4) informal autobiographical non-fiction. The final included extracts were selected from the following texts:

Group A:
(1) The Memoirs of Sherlock Holmes (Doyle, 2012).
(2) Eleanor Oliphant is Completely Fine (Honeyman, 2017).
(3) Exploring Autism: A Conversation with Uta Frith (Burton, 2013).
(4) Freedom to be Honest – an article from Your Autism Magazine (Packham, 2017).

Group B:
(1) Great Expectations (Dickens, 2012).
(2) Faith and Hope Go Shopping (Harris, 2010).
(3) How Selfish is Your Search for Happiness? – an article from The Psychologist magazine (Smith, 2018).
(4) Expert Interview with Gretchen Rubin on Finding Happiness (2018).^3^

### 2.4. Procedure

Prospective participants completed the screening process via Qualtrics. The process included the informed consent procedure, a demographic questionnaire, the QT and the AQ. Participants who screened out or did not choose to enroll in the subsequent study had their data removed. Informed consent was obtained at three points (1) before the screening process, (2) before the reading tasks and (3) before the follow-up interview. During each stage, participants received both a university standard information sheet and an easy-read version which avoided complicated explanations and used clear photographs and text segmentation.

Following the informed consent procedure, participants were provided with the 8 short text extracts as digital text documents, alongside corresponding audio files of the third author, who is a trained reader, reading the texts aloud. The texts were split into part A and B, with the texts numbered from 1 to 4 within each folder, in the numerical order shown in Section “2.3. Study materials.” Participants were asked to complete the texts in order, starting with part A. Eight participants read Group A texts first, with five starting with Group B texts. The reading order was alternated in this way to try and control for any order-specific reading outcomes. Participants were instructed to listen to the corresponding audio file while reading each text in full for the first time. For each of the 8 extracts, participants were asked to complete a short questionnaire which asked them to: (1) point to the most literary (higher quality) part of the text, (2) highlight the part of each paragraph that felt most important, (3) explain what they felt they had got from reading the text, (4) identify a part that baffled them and explain why, (5) identify a part that caused them to feel something and explain why, (6) add in any additional, overall thoughts and (7) note how many times the text had been read and listened to.

Once parts A and B had been returned, participants were then invited to a follow-up interview with the first author. During interview, the researcher chose a highlight from question 2 for each of the 8 extracts, which was then read aloud to the participant for re-immersion. Participants were then asked to further expand on what stood out about this part of the text. Participants were then asked to pick a second highlight for each text that they would most like to discuss. Additionally, participants were asked some questions about their wider experience of the study methods and specific texts used. Upon return of the reading data, participants were reimbursed with a £10 Amazon voucher for their time. Participants who took part in the follow-up interview were reimbursed with a further £10 Amazon voucher. Two autistic participants were interviewed in person, in a quiet university interview room. All other participants were interviewed through Skype or Microsoft Teams, with two (both non-autistic) electing to take part using audio only and the remaining eight taking part via video call. Interview audio was recorded using dictaphones and later transcribed for further analysis.

The first author is trained to Master’s level on semi-structured interviewing and conducted all of the final interviews with no other researchers present. All autistic participants were made aware that the interviewer would be an autistic adult. The researcher was acquainted with two of the autistic interviewees and had previously interviewed an additional two autistic and two non-autistic participants from previous, related research projects.

### 2.5. Analysis

SPSS (IBM statistical package for social sciences) was used to organize quantitative demographic data and to calculate descriptive statistics.

Interviews were transcribed using edited transcription, where irrelevant false starts, filler sections and areas of repetition were omitted, unless used to convey importance or significance. Aside from these instances, participants’ words were transcribed verbatim. All transcription was completed by the first author, who has prior experience of interview transcription. Resultant transcripts were not sent back to participants as there were no areas in need of further clarification.

A form of literary close reading analysis (Billington et al., 2019) was chosen as the primary analytical approach in order to inductively explore psychological shifts within participants as a result of their reading. This analysis relies upon the language of readers as a “main point of access to moments of subtle mental change,” giving researchers access to the “imprints” of reading (Kaszynska, 2015). The close reading practised in this context is in the tradition of “practical criticism” founded by Richards in 1929 (Richards, 2017), which emphasizes analytical attention to the words on the page, without preconceptions about their meaning. Reflexive thematic analysis (Clarke and Braun, 2014) was additionally used to deductively analyze data relating to the study method and texts used. Analytical stages were as follows:

1) The first author transcribed all interviews to achieve data immersion, marking areas of initial literary interest. The second, third and fourth authors reviewed data from 5 participants for immersion, marking further areas of literary interest. Of these 5, 4 autistic participants were chosen due to the autistic data being richer than the non-autistic data.
2) The first and second author agreed on initial themes and discussed these with the wider team until the themes had been agreed.
3) The first author applied a line-by-line analysis to all data, re-adjusting themes from stage 2. Findings were sent to the wider team with data examples to illustrate the themes and subthemes.
4) The second author reviewed the findings from stage 3, re-analyzing any areas of uncertainty.
5) Resulting themes were then deliberated by the team, with theme names and framings adjusted to capture the main elements of significance within the themes.

## 3. Results

### 3.1. Summary of reading-aloud design findings

Overall, 6 participants (3 autistic) liked having the pre-recorded reading aloud files, while 4 (2 autistic) disliked their inclusion and 3 (2 autistic) felt there were both positives and negatives of having them available. Regardless of participants’ opinions on the reading aloud files, there was a sense across all participants that listening to the reading aloud files while reading the texts themselves slowed them down. Most readers preferred to read at their own pace without audio, but where readers found themselves struggling to immerse in a text, they often felt the audio helped by slowing them in a way that prevented attentional difficulties. By contrast, most readers across the two groups found it difficult to listen to the texts that they otherwise did feel immersed in, due to feeling that this created distraction.

### 3.2. Qualitative analysis results

The final analysis (see Table 2) comprised 3 themes: (1) From Surface Reading to Intuitive Engagement, (2) Imaginative Feeling and (3) Going Forward from the Reading Experience. Quotes are spilled by neurotype (A, autistic; N, non-autistic) and the text that participants read. Where quotes came from the later interviews, a note is made of this. Within participant quotes, words that highlight important thinking in relation to the subtheme are highlighted in bold.

**TABLE 2:** Themes and subthemes.

From surface reading to intuitive engagement	Imaginative feeling	Going forward from the reading experience
External reading	Feeling for the text	Unaware of own abilities
Getting into the text	Feeling from the text	Resulting salience
Uncovering deeper contexts	More than one	

#### 3.2.1. From surface reading to intuitive engagement

##### 3.2.1.1. External reading

Each reader experienced times where they remained on the outside of some of the texts, struggling to get into a text and to feel within it. During these times, readers tended to summarize the text based on surface-level appraisals. This often resulted from a sense that the text had not provided room for imaginative feeling:

*(P12A: Gretchen Rubin) “the author is **telling us** that life is what we make it.”*

*(P40N: Gretchen Rubin) “practical **advice** on how to take control of your own happiness.”*

This was a common issue across readers for the non-fiction texts. As highlighted in participant 12’s quote, these texts tended to “tell” the readers about something, giving them key information to take away rather than encouraging them to emotionally discover it for themselves. While the fictional texts did provide this room for imaginative feeling, readers did still experience times of struggling to get inside the fictional texts:

*(P19A: Sherlock Holmes) “not entirely sure what exactly I **could** have gotten out of it because I was more committed to **trying to understand** the text”*

*(P30N: Great Expectations) “**Shows** that Pip is a commoner and Estella looks down on him.”*

Here, participants 19 and 30 experienced difficulty getting into the texts as a result of their own concern with objectivity. For participant 19, there was a self-conscious focus on wanting to understand what should be taken from the text, rather than exploring the text intuitively and gaining from it through his own feelings. Similarly, for participant 30, the focus is on summarizing the interaction between Estella and Pip, in a way that reduces the feeling down into something more objectified, less complex and less felt. Across readers, surface reading was a more common barrier for the classic literary texts as compared to the modern literary texts. This appeared to be due to concerns amongst readers about having “correctly” understood the content of the classic literature.

##### 3.2.1.2. Getting into the text

Readers often tried to get on the same wavelength of a text by constructing visualizations of the scene, enabling them to feel a sense of actively being inside the text. While this demonstrated an intentional desire to immerse within a text, it was sudden moments of unexpected feeling that surprised readers into a live reality to immerse in:

*(P4A: Sherlock Holmes: Interview) ““I’d come to believe that he was an orphan with no relatives living. And 1 day he began to talk about his brother.” It **strikes me** that they weren’t particularly good friends if they did not ask that”*

*(P28N: Eleanor Oliphant) “**I** think something that **struck** me is her interaction at the bar – as a reader **we** cringe”*

For participant 4, this shock from the text comes not from reading it in the original moment, but by reciting a quote to bring the text alive once again, recreating the sense of shock. This enables the participant to go deeper inside the mind of the text, thinking beyond the basic context provided to further infer something about the relationship between Holmes and Watson. For participant 28, the experience of shock while reading resulted in an emotional opening up to feel with the minds in the extract, which in the shift from “I” to “we” further resulted in a move to consider the minds of other imagined readers too.

Once readers had successfully got inside a text, they began to trust their own instincts while reading, rather than focusing on concerns about what they should be taking from the text. Readers initially showed this by pointing to subtleties in the language itself that provided a window into deeper implied subtexts:

*(P12A: Eleanor Oliphant) “A structure of sentence that wouldn’t be perceived as normal to most ears.”*

*(P38N: Eleanor Oliphant: Interview) “I wouldn’t really use full sentences when ordering a drink.”*

During these moments, readers were not yet doing something with the language to uncover deeper meanings, but were identifying significant moments where something deeper might be going on. This led readers to start thinking through the complexity of the texts in a way that uncovered some of the subtext beneath the immediate language:

*(P8A: Eleanor Oliphant) “I **don’t feel** baffled by any of it**, but** I am rather intrigued about how Eleanor has ended up in this situation **given that** she **seems not to want** to be there.”*

*(P40N: Eleanor Oliphant) “why has she never been to a pub before and why does she use such formal language in an informal environment?…[added during interview] What’s happened **before**?”*

Participant 8 had started to engage with live thinking about the text in a way that starts to explore how Eleanor might have been feeling. Similarly, participant 40 questions the immediate subtext, starting to think about an imagined past for Eleanor in a way that makes her a more real mind to understand through live thinking.

From these explorations, readers themselves started to identify the importance of having room to infer and feel things for themselves:

*(P8A: Eleanor Oliphant) “the use of words here seem very carefully chosen to allow the reader to **infer** a lot about the inner life of the narrator, **without doing anything so heavy-handed as telling** the reader what the narrator is like”*

*(P38N: Faith and Hope) “Describing how it is to experience old age and the diminishing of dreams well **without stating** this exactly”*

It was the being *allowed* to think about inner lives that participant 8 points to which enabled readers to more readily immerse in the fictional texts. This contrasted to being *told* things directly in the non-fiction texts. Where the fiction texts had started to become a live reality to feel inside, the readers were left wanting to read more.

##### 3.2.1.3. Uncovering deeper contexts

Once a reader had got inside a particular text, they were then able to get into a rhythm of using their own intuition more fluently to unpick deeper subtext. In interacting with a text in this way, readers were better able to unpick the contextual depths held within it by thinking about its contrasts:

*(P10A: Faith and Hope) ““unsuitable, it may be,” because I like the **reframing** of the term “unsuitable” from something that causes Faith anxiety to something Faith regards as **the label of another”***

*(P40N: Sherlock Holmes: Interview) “he was **kind of** lacking **something** in a kind of social…**yet, in other ways**, he excelled…it was the fact that **whilst** he was kind of like, we say preeminent and like quite an impressive person, if you like, he still had kind of flaws of his own really”*

Readers were then able not only to point to important parts of the fictional literature, but to explore the bigger feelings and meanings that were held within small literary moments:

*(P8A: Great Expectations: Interview) “if that paragraph had stopped right there, at the thought of being ashamed of my hands before, it **contains within it** the meaning of itself, which is I haven’t been ashamed before…now he is ashamed”*

*(P30N: Faith and Hope: Interview) “It was only a small sentence of just saying “you’re wrong,” like that would make all the difference. Just that **one small sentence can like make a big difference**”*

By starting to explore this complexity which was contained within the ostensibly simple, readers were then able to intuitively explore the complexity of feelings for characters within a text:

*(P8A: Sherlock Holmes: Interview) “when he says strange, **he means** something that’s had a very big effect on him. So, I think **it suggests** that there’s a big backstory there that **he is hinting at**, with this very general statement that he doesn’t want to talk about **just yet**”*

*(P40N: Faith and Hope) “**even though** she could not afford the shoes, the act of kindness with the rose gave Faith a **moment** that she **continues** to cherish”*

In the above examples, Sherlock and Faith have become real minds for the readers. They are able to feel with and think through these human minds in a way that results in these complex considerations of deeper meaning for the characters, beyond what is immediately available in the text.

These in-depth explorations were specific to the fictional texts and occurred for both the classic and contemporary literature. For the non-fiction texts, there was more of a deconstruction of the texts by the readers as opposed to emotionally getting inside them. This deconstruction came from a sense that there was something missing, or a deeper intention within the text that was hidden by the surface information available to the readers:

*(P19A: Gretchen Rubin) “One thing **I felt that was lacking** was that the author did not elaborate on how her successful improvement in happiness helped her in life”*

*(P30N: Uta Frith) “it might be **a bit reductionist**, feel like there is more to autism than just lacking this innate ability”*

#### 3.2.2. Imaginative feeling

##### 3.2.2.1. Feeling for the text

Immersion in a text also allowed readers to feel through it to varying levels. While readers were not always able to feel *with* the minds contained within a text, they were often able to feel *for* them:

*(P8A: Chris Packham: Interview) “**poor Chris**, he can’t just learn a set of rules and figure out how to follow them because the rules aren’t written down anywhere”*

*(P40N: Great Expectations) “finally when left alone the impact of this torment and how Estella had made him despise himself all came to the surface. It **made me** feel sad **for** Pip”*

This experience of feeling sorry or sad *for* someone within a text was experienced across readers but more commonly by non-autistic readers. This was because autistic readers more often felt *with* the people inside a text as opposed to feeling *for* them. A surface feeling for minds held within a text tended to result when readers related to an experience on its surface:

*(P12A: Chris Packham) **“I can relate to** this, I work with people all day because I have to.”*

*(P38N: Great Expectations) “Pip was described as crying from what I perceived as an unnecessary feeling of shame brought out by Estella’s bullying. This **can be related to** my personal experience of being put down and invokes empathy”*

This surface relating to something created a sense of familiarity, where feeling for a person in a similar situation was easy. The lack of surprise at being able to feel for these experiences prevented deeper feelings of engagement with the minds in the texts.

This ability to feel for a text, its situations and characters, was found across fiction and non-fiction extracts, but tended to apply more to the fictional texts. Where feeling was evoked by non-fiction, this was more for the autobiographical texts than the explanatory, third-person extracts. Where the non-fiction texts addressed human feeling, there was often an attempt by readers to prescriptively apply empathy, rather than a feeling emerging organically toward the people in the texts:

*(P12A: The Psychologist) “empathy helps us understand one another and potentially treat each other better”*

*(P26N: Chris Packham) “Autistic people speaking out about their experiences is needed to help other people understand what it is like to be autistic. This may then lead to positive behavioral changes in the wider community that will help people with autism.”*

Here the participants were trying to *apply* empathy due to a sense that they ought to do so within the context of the texts. This came from a sense, as described by participant 12, that empathy is a helpful instrument to deploy. The difficulty with this attempt to empathize with texts was that there was no sense of the readers having been moved into feeling for another person within the text. Therefore, this more systematic approach to feeling meant that empathy was seen as something that can and should be deployed, rather than something that needs to spring and grow organically from spontaneous feeling. As demonstrated by participant 26, this led to difficulties for non-autistic readers in trying to feel for autistic people represented within the non-fiction texts. For participant 26 there resulted a shift in blame and responsibility for behavioral change from autistic people onto non-autistic people. The result is then that the reader maintained the artificial binary categorical differences between autistic and non-autistic people, rather than experiencing a collapse of these differences to feel with the imagined minds of autistic people as similar Others.

The initial move from feeling for to feeling something closer to an authentic empathic experience came from readers feeling difficult feelings for a character. All fictional extracts dealt with human disadvantage in a way that prompted readers to feel for character experiences. However, *Eleanor Oliphant is Completely Fine* (Honeyman, 2017) in particular led to difficult feelings for the characters within the extract, due to the consistent lack of mutuality during social interactions between characters within the extract:

*(P12A: Eleanor Oliphant) “I find the directness of the sentence **makes me uncomfortable**, in that it **could almost be confrontational** but also find that the language used **doesn’t sit well** with me. I think it does this because I can understand using these words in this manner and actually, it’s **my own experiences** in the world that have shown me that I can’t structure sentences like this without antagonizing people”*

*(P28N: Eleanor Oliphant: Interview) “as a reader, you’re just thinking “no!”…She’s done so well, but then sort of it just **makes you cringe a little bit**”*

The sudden “no!” from participant 28 highlights the involuntary feeling with Eleanor that had started to come out of feeling for her by cringing at the social encounter. These were commonly occurring feelings toward Eleanor for non-autistic readers. By comparison, autistic readers, such as participant 12, did feel uncomfortable for Eleanor, but did so from the perspective of having experienced similar situations themselves. Therefore, for the autistic readers the feeling was less about surprised compassion and more about feeling with Eleanor through the evoked difficult and personal memories that were then re-experienced and re-interpreted with the text in mind.

##### 3.2.2.2. Feeling from the text

As readers became more immersed, they started feeling *from* the texts they were reading as well as feeling *for* them. This came from spontaneous feelings being unexpectedly evoked through the reading process. For autistic readers, there sometimes emerged a shared feeling between the text and themselves, enabling them to feel together *with* the fictional characters:

*(P4A: Eleanor Oliphant: Interview) “**I struggled a bit** with this one, I had to read it more than once. And I think **it was kind of that she struggled** with it”*

*(P10A: Great Expectations: Interview) “I think **I felt similarly to Pip** in that one in that I didn’t really…I don’t think I understand fully the implications of everything that was going on”*

While these examples show some sense of difficulty with the text, the participants have been able to hold onto these difficult shared feelings to remain emotionally immersed, rather than reverting to surface appraisals. What results is a powerful sense that the readers have not only developed a sense of empathy toward the text, but found empathy for themselves within it.

Part of what moved autistic and non-autistic readers to feel *from* the text was a move from basic relation to a text to a more surprising, felt relation to the emotional experiences within it. This took readers beyond easily relating to something, instead being moved by how strongly and unexpectedly they had found something that felt true to themselves within the extract:

*(P7A: Eleanor Oliphant: Interview) “I’ve kind of done this before…maybe in social situations where I’ve been a bit “no, I’m going to do it like this. Don’t question me”…And I just read it and I was like **“oh God, that’s you.** You’ve done that before. **Oh no**” It was like that actually really, peculiarly affected me”*

*(P25N: Gretchen Rubin) ““I wasn’t depressed, and I wasn’t having a midlife crisis, but I was suffering an adulthood malaise – a recurrent sense of discontent, and almost a feeling of disbelief.” This part **made me feel like it was me talking**, there have been times in my life when I have felt **exactly** like this”*

For autistic readers, these moments of felt relation were often painful, as shown through participant 7’s sudden revelation of *“Oh God, that’s you.”* This moved autistic readers to relive memories of their own, using their new perspective as readers of the evocative text to reassess themselves through the recollection of relevant memories:

*(P4A: Gretchen Rubin: Interview) “I **enjoy things retrospectively**. So, with my anxiety, sometimes I don’t actually enjoy what I do. But then **when I think back to it, I enjoyed it** in retrospect…**the memory of it**”*

*(P10A: Gretchen Rubin) “when life was taking its ordinary course, it was hard to remember what really mattered; if I wanted a happiness project, I’d have to make the time,”…**I don’t think I experience it quite the same as others**. Often, for me, the “ordinary course” of life **brings happiness in itself, in its mundanity**”*

Having felt from the text as well as for it, readers were then better able to imagine how the minds within the text might be feeling during emotional situations. The to-and-fro of feeling between readers and the texts led to more complex assessments of feeling amongst fictional characters in particular:

*(P10A: Great Expectations) “**He is unsure** what to say and what to do, and when he does attempt to say and do things he is met with **reactions that assure him that they were the wrong things** to say and do; he is then **so overwhelmed by it all that he breaks down a little bit**. This was how many of my attempted social interactions went when I was younger, and how things still go sometimes today.”*

*(P30N: Eleanor Oliphant) “**she didn’t realize** why she was being rude, **she thought** she was just asking a question. **But to the barman** those questions would have seemed rude and sarcastic”*

For non-autistic readers, the complexity of perspective that came from this imaginative feeling led to the readers starting to think about multiple competing perspectives, as participant 30 is doing between Eleanor and the barman. While autistic readers were similarly able to feel for competing perspectives, they also engaged with self-reflection through these complex feelings in a way that enabled them to continue feeling in company with the text. In this way, autistic readers were not only moving between the inner perspective of a main character to the outer perspective of a secondary character, but also started to shift from their own feelings that had come from a text to how an imagined, outer perspective might think or feel about this:

*(P4A: Eleanor Oliphant) “there probably weren’t aspects in her that I recognized in myself, although, probably externally, **other people would say I’m very similar…I wouldn’t say I felt that connection**”*

*(P8A: Great Expectations) ““I had never thought of being ashamed of my hands before” This made me think of **occasions when I’ve viewed myself “through someone else’s eyes” and suddenly been ashamed** of something about myself”*

By being able to move between the inner feelings of the characters, their own inner feelings and the imagined perspectives of someone viewing them in the midst of these feelings, autistic readers showed a stronger sense of resonance with the texts:

*(P7A: Eleanor Oliphant) “I felt quite **in tune with** Eleanor, so I guess the extract as a whole just affected me, as it made me remember situations in which I’ve acted in the same or similar ways”*

*(P10A: Faith and Hope) “I felt the knowledge **clang deep** in my insides, like something falling down a well.” – This **rang particularly true** to me, as it’s something I’ve felt often.”*

This musical language, such as “*in tune,” “clang deep”* and *“rang true,”* was very common amongst autistic readers but was not used by the non-autistic readers. The language represented a sense of readers feeling a sense of “attunement” between their own feelings and the feelings of the text. In this way, autistic readers often achieved a strong synchrony of feeling between themselves and the texts, enhancing their immersion and what might too easily be called “empathy” toward and from the texts.

##### 3.2.2.3. More than one

From the complex consideration of inner and outer character perspectives, readers moved toward feeling for multiple characters at the same time. For two autistic readers and one non-autistic reader, this led to a rethinking of the text, moving from their initial impressions through the mind of the main character, to incorporating feelings for more perspectives:

*(P10A: Eleanor Oliphant) “I simply could not fathom why he was making such a fuss about it”…“I agreed **at first**, **then** thought that **perhaps** Raymond felt the same way as Eleanor about unfamiliar situations”*

*(P40N: Great Expectations: interview) “the thing that stuck with me on this was, and I’ve kind of thought about this a little bit more actually, so I’ve kind of made out previously…that like Miss Havisham was like the bad guy and that, actually. Estella and Pip are obviously the victims, even though Estella’s being mean to Pip. But actually, I could probably **take it a step back** and say that…Miss Havisham probably isn’t a bad person either, **actually**…I put this little thing about she’s doing all this manipulation for her own kind of wicked kind of self-gratification, which is probably true. **But** she’s obviously been harmed in some way, hasn’t she, previously? **Although** the way that she’s kind of dealing with this is not healthy, and it’s impacting on other people, I think that they’re probably all victims in some sense, and it’s almost like it’s kind of self-destructive for all of them, in a sense…some people got more say in it than others’*

Participant 10 has been able to rethink an initial alignment with Eleanor’s own thoughts, to further feel with Raymond as well by carefully contemplating how he might be feeling in the same situation. For participant 40, there was a move beyond summarizing Miss Havisham as *the bad guy*, toward feeling with her through an imagined past whilst also accepting that her intentions could still be *wicked* and feeling for her regardless of the difficulty her intentions add. This immersed thinking and feeling inside a text also led readers to hold multiple emotions within themselves from the texts:

*(P7A: Faith and Hope) “I feel are **uplifting, but at the same time tinged with sadness** as you know that Faith and Hope have had a wonderful adventure but must now go back to their “real life””*

*(P28N: Eleanor Oliphant) “**So many** emotions – **firstly**, you’re hopeful Eleanor will reach out to her colleague on an emotional level. **Then** you **start to** cringe and feel disappointed for her colleague. **You also feel** that Eleanor is trying to connect and be reasonable by saying it can wait. And then the **final** “extravagant” – as a reader it made me laugh, **but also** wince a little bit”*

#### 3.2.3. Going forward from the reading experience

##### 3.2.3.1. Unaware of own abilities

While autistic and non-autistic readers engaged with reading in similar ways, what the readers took from the reading experience varied between the groups. For autistic readers, there was a sense that they were previously unaware of and thus surprised by their abilities as readers and more generally as empathisers. For example, participant 12, when reflecting on his differences as an autistic person tended to make statements that overlooked the socio-emotional skills he had exhibited through his reading:

*(P12A: Uta Frith): “So much of my life has been based on what is basically pre-prepared scripts, being **caught out by something I’m not prepared for** is like having the ground open up under my feet…I really can’t comprehend multi-tasking thoughts.”*

The overall difficulty for this participant was an abiding sense of his self-described “difficulties,” rather than looking at what was achieved through the struggles that occurred. Where the participant saw himself as struggling with the unexpected and feeling the strain of multi-tasking, his reading showed that he engaged more emotionally with a text, as well as being able then to hold onto more than one complex thought or feeling. These difficulties for participant 12 in understating his abilities seemed to stem from a prior sense of inferiority, including the feeling that he could not often be his *true* self in the normal social world:

*(P12A: Chris Packham) “I much prefer my own company and used to walk off into the hills of Kintyre when I was a teenager, miles of countryside **without another person to be seen, I felt at peace there**. There are still **very few people I can be 100% myself with**.”*

What had been achieved through his reading was a closer sense of this *true* self he described. In this way, the texts were able to act as a social simulation for the reader, creating a social environment that was more enabling.

Similarly, participant 19 was often focused on his struggles while reading, highlighting what he had found difficult:

*(P19A: Great Expectations: Interview): “in terms of **attaching the emotion** to it, it’s **not easy for me** to think of an emotion to attach to it….but in terms of, if you want me to do that now, it’s **hard for me to think about that**, because I feel that, obviously, you know, you’re been criticized **right from the offset**, and I feel that that’s something which is something I don’t think that anyone likes really.”*

However, even in thinking more about his difficulty here in naming or labeling an emotion, the participant becomes more comfortable in holding onto the intangible feelings he does have. From here, he is able to start to think about the feelings as part of a situation, beyond a single and nameable emotion. Importantly, this is what the literature is requiring of the readers, for them to stay with the host of intangible feelings as Pip had done within the text. When the participant managed to overcome these concerns to get inside a text, what resulted was a depth of understanding toward the text that came out of the participant’s own intuition:

*(P19A: Faith and Hope) “This caused me to **feel something** because I could appreciate that **Faith’s disappointment** in not being able to get the shoes that she wanted has been **restored somewhat** in the generosity of the store assistant trying to do something to give Faith **something to remember** the day by.”*

For participant 19, his lack of confidence in his abilities appeared to stem from a sense that his struggles to fit into society had resulted in felt disability through not having been accommodated by others. This itself was something that he was able to start exploring through his reading experience:

*(P19A: Great Expectations) “This part of the text **made me feel something** because having also had a **difficult upbringing** in not knowing from the beginning that I was autistic and **not having the adjustments** that were made to me in a neurotypical world made me relate to Pip’s story.”*

As a result, participant 19 started to see the value in literature, through its ability to enable a reader to feel human realities, through a simulation of the world, in a way that more formal disciplines and programmes could not:

*(P19A: Interview) “I don’t relate very good to reading fiction…but what it’s taught me is that there are things that you can relate to, when reading fictional literature. And there are certain situations that they talk about that, you know, the **only other way** you experience that is in say, everyday life.”*

##### 3.2.3.2. Resulting salience

Across autistic readers, there was a holding onto characters and situations within the texts as imagined real human beings and experiences to refer back to, and not just explicate. This became a helpful way for these readers to express themselves, particularly when the readers struggled to think of an easily recognized adjective to describe their own feelings while reading:

*(P7A: Great Expectations: Interview) “**I felt an emotion** with that, that I didn’t feel in the rest of the text. And **I felt that Pip there** was really kind of **battling with his emotions**. But he didn’t… it was like an **inner turmoil** and he couldn’t kind of deal with **he couldn’t identify his emotions and deal with it himself. And I kind of identified with that**.”*

*(P19A: Eleanor Oliphant: Interview) “In terms of how that made me feel, though, yeah, it wasn’t really…it’s **hard to put a feeling on it**. But I would say that I just felt, again, like I could empathize with **somebody** like that…So, it just made me feel something in a sense that, yeah, we’ve been there before at times…reading this now makes me think, ‘oh, I can relate to that **situation**.”*

Where autistic readers tended to think about detailed mentalities, non-autistic readers tended to reduce their reading experience down into messages, ideas or feelings as opposed to taking away a sense of a complex person to think about and feel back through. For example, participant 28 had been a very immersed reader throughout, but tended to rest on “key” ideas about how she felt she should or should not think about autistic people:

*(P28N: Eleanor Oliphant: Interview) “it’s never explicitly said anywhere [that she’s autistic], but just as a reader, you automatically just start kind of making those connections. But should we? Is that kind of not unfair, that we just sort of stereotype people in that way?”*

*(P28: Chris Packham) “as a society, we need to look at maybe the positives of things like autism. You know, I think it’s so easy, like I said, to come up with the lazy stereotypes of kind of, I don’t know, Rain Man, or someone who’s great at computers or something. And I think you might say we kind of lean toward those lazy stereotypes.”*

Through her considerations of whether it was right to automatically stereotype Eleanor and how people might stereotype people like Chris, there is a resultant consideration about how to think about autistic people in everyday life. In storing these key thoughts for wider application, the holding of Eleanor and Chris as complex minds to continue thinking and feeling through becomes something helpful to day-to-day socialization. While these applications might prove beneficial, what was lost for non-autistic readers was the ability to continue holding onto complexity, as they had in their reading, for further use in day-to-day social interactions. Where autistic readers were often comfortable in holding onto uncertainty and intangible but relatable feelings, non-autistic readers appeared to prefer clarity, drawing conclusions in order to reduce the information being held as much as possible.

## 4. Discussion

### 4.1. Summary of findings

The study aimed to (1) examine differences between text types within a reading aloud design involving autistic and non-autistic readers, with a specific focus on comparing serious literature with non-fiction and (2) investigate whether texts aligning with autistic experiences could enhance the reading experience for autistic readers, and whether there would be any resultant understanding for non-autistic readers. Findings are discussed in Sections “4.1.1. Challenging theoretical assumptions of an autistic empathy deficit” through “4.1.3. Inclusive shared reading designs” in relation to previous theoretical assumptions and research.

#### 4.1.1. Challenging theoretical assumptions of an autistic empathy deficit

The complex, felt responses toward the texts in this study amongst all readers challenges the E-S theory view that autistic people experience a broad empathy deficit when compared with their non-autistic peers (Baron-Cohen, 2002, 2008). Instead, the autistic readers in this study were more likely to share the emotions held within a text. Although it could be argued that this reflects egocentrism (Lombardo and Baron-Cohen, 2011; Bodner et al., 2015), the shared feeling came from a sense of attunement between readers and the minds within a text. Therefore, the perspective-taking involved and resultant feelings felt more two-way, with readers accounting for difference as well as similarities between their own perspectives and the imagined minds within the texts. This supports the idea that moving parts of a text extend beyond an author and the resulting text, to become part of a reader (Barnes, 2018; Limburg, 2021). The ability amongst autistic readers to more readily feel with a text tended to result from the ability to not only move into literary perspectives, but to also imagine themselves in the midst of embodying the mind of a character from an imagined outside perspective. This complex mobility of perspective further challenges the idea that autistic people possess a deficit in their ability to take perspective or embody other minds (Baron-Cohen, 1997, 2008; Lombardo and Baron-Cohen, 2011). The complex depth of feeling for fictional minds that has been demonstrated here by autistic readers instead supports the idea that autistic people may experience a greater depth of feeling as a result of attending more to detail (Happé, 1999; Hill, 2004; Murray et al., 2005; Murray, 2020). However, the mobility of perspective showed here challenges the view that this depth of feeling comes at the expense of understanding social breadth (Happé, 1999; Murray et al., 2005).

Additionally, results here support earlier findings in showing that autistic people are more likely to evaluate themselves through an imagined third-person perspective (Schriber et al., 2014; Burrows et al., 2017; Arnaud, 2022). The clarity that this study adds is that this third-person view of self is not simply a systematic attempt to gain objectivity, but rather a more felt and complex insight into themselves. Current findings also support the idea that the tendency for third-person perspectives may result from self-consciousness amongst autistic people in relation to their own abilities (Schriber et al., 2014). The autistic readers in this study underestimated their abilities as readers and more generally as empathisers in a way that contradicted their demonstrated abilities. This self-consciousness appeared to have been learnt through a lack of accommodation within wider society, highlighting a further need to challenge stigmatizing views of autistic people (Green et al., 2005; Pearson and Rose, 2021). In line with this, there is an additional need to review education across society in terms of what it means to have “emotional intelligence,” so that the socio-emotional abilities of autistic people are not reduced down and viewed as deficient in comparison to what is assumed to be typical socio-emotional processing. Findings here further emphasize the value of reflective reading as a more open method to understand autistic social experiences in a way that moves away from deficit views (Chapple et al., 2021a,^2022^). In this study, the serious literary texts enabled autistic readers to engage as a truer, less self-conscious, version of themselves once they were fully immersed. This further highlights the value of literature in unlocking the potential of a reader’s inner self (Farrington et al., 2019; Davis and Magee, 2020) and shows the personal value for autistic readers.

#### 4.1.2. Exploring social differences between autistic and non-autistic readers

In the current study, both autistic and non-autistic readers were able to read in similar literary ways that engaged them in imaginative ways with the depth of feelings held within the texts. What did differ between them was how they cognitively stored the social data from the texts for later potential use. In line with suggestions from the WCC and monotropism theories (Happé, 1999; Murray et al., 2005; Murray, 2020), autistic readers were more likely to attend to and hold on to the detail of a text. Therefore, autistic readers, enabled by the literature, tended to hold onto the intangible, literary moments beyond the reading experience. This further emphasizes the ability of serious literature to encourage a holding onto the intangible (Farrington et al., 2019), while building on previous findings (Chapple et al., 2022) to show that autistic readers may continue to be more literary-influenced in ways that go beyond the immediate reading experience. Importantly, the reading experience enabled autistic readers to hold onto complex detail in a way that did not result in them feeling overwhelmed or having difficulties understanding broader contexts (Happé, 1999; Hill, 2004). This was achieved through maintained representations of characters as felt people who could hold complex thoughts and feelings. In this way, autistic readers could then re-ignite literary complexities by drawing on the character.

By contrast, non-autistic readers did not tend to hold onto characters as real people to think about and feel back through. Rather, non-autistic people tended to extract core ideas or feelings for later use or reflection, by a form of data reduction. This further highlights the double empathy problem (Milton, 2012) in suggesting that autistic and non-autistic people may have differing social norms. Specifically, non-autistic people appear to extract core information that reduces complexity down, meaning it can be easily accessed and generalized later (Lombardo and Baron-Cohen, 2011). This ready competence for data reduction contrasts with autistic people, who appear to instead favor holding complexity in a way that would encourage slower, more careful considerations of new social situations without pre-emptively applying “core” knowledge (Milton, 2012; Chown, 2014; Chapple et al., 2021a,^2022^). Ironically, this means that non-autistic people take what the E-S theory would call a more systematic approach to social learning (Baron-Cohen, 2002, 2009). This both challenges the argument that systemizing is not conducive to empathy and the view that it is autistic people who are more robotically systematic (Baron-Cohen, 2002, 2009). Each approach by the two groups offered different advantages: the systematic approach offering brevity and the more complex approach offering complex understandings that were more natural and synchronous. However, the contrast in these approaches would likely result in difficulties establishing mutuality for social reciprocity, as suggested by the double empathy problem (Milton, 2012). What this means is that reading alone is unlikely to aid an overcoming of the double empathy problem, even when contemplating serious literature or material explicitly exploring neurodivergent experiences. Specifically, when non-autistic people were reading the texts that depicted autistic experiences or the double empathy problem, there was often an attempt to deploy empathy in a systematic way that failed to get them immersively inside the text. This contrasts to previous findings, where non-autistic readers reading together with autistic readers were better able to hold onto complexity with their autistic reading partners, in a way that overcame the double empathy problem (Chapple et al., 2021a). However, it remains unseen whether non-autistic readers from this study would be able to recall their reading alone experiences to re-activate the complexity of the texts they had read.

#### 4.1.3. Inclusive shared reading designs

The use of audio files of texts being read aloud overcame concerns with being read to amongst autistic readers (Chapple et al., 2021b). However, the use of pre-recorded readings did not result in the sense of liveness that is important in creating openness and a sense of connection for readers (Longden et al., 2015). Although the method used here was unable to capture the full value of reading aloud designs, readers did still engage with and benefit from the serious literature in particular. Texts were particularly beneficial and more readily immersed in where the social reality inside the text created uncomfortable or surprised feeling within a reader, often also registered by increased syntactic complexity and a more powerful vocabulary for the emotions. This supported the idea that texts dealing with human adversity, and promoting difficult feelings as a result, may result in a greater sense for readers of having been creatively moved (Strick and Van Soolingen, 2018; Davis, 2020). Findings here that surprised relatability to a character or situation was moving to the readers contradicts earlier findings that autistic people might need to read texts that are directly relatable to their lived experiences to achieve maximum immersion (Chapple et al., 2021b). Rather, easily recognized experiences that evoked unsurprisingly familiar feelings failed to shift readers out of default ways of thinking in the way that serious literature can (O’Sullivan et al., 2015; Farrington et al., 2019; Davis, 2020). While previous work has suggested that the age of classic literature can provide a sense of surprised relation through a somewhat unfamiliar language (Farrington et al., 2019), the classic literature used in this study tended to instead promote self-conscious concern with having correctly understood the older language. Therefore, contemporary literature (2010–2020) may offer an initial alternative way to get less confident readers used to trusting their own intuition, before working up to older works that may represent less easily understood norms and ideas. However, all readers showed an increased immersion while reading serious literature compared to the non-fiction texts. While readers engaged more with the autobiographical non-fiction, these texts still prompted a sense that any socio-emotional subtext was unobtainable due to a lack of room for imaginative feeling. These findings support the idea that directly autobiographical writing fails to capture the harder-won but more deeply felt autobiographical elements that indirect and even fictional works can hold (McCartney, 2021). Although earlier findings have shown that autistic people can find emotional value in reading non-fiction (Chapple et al., 2021b), current findings demonstrate that serious literature offers the most advantage for both autistic and non-autistic readers in encouraging deeper self-other reflections.

### 4.2. Limitations and future research

Findings from the current study are limited in their generalizability to autistic and non-autistic people in wider society. Firstly, all participants were educated to GCSE level or above. This was likely a result of the self-selecting nature of the recruitment method, where participants had to be willing to read multiple short texts including serious literature. Additionally, the fact that participants were willing to reflectively read the texts indicates that they may have been more willing to think reflexively about serious literature (Chapple et al., 2021b). Together with the inclusion criteria requiring participants to not have a reading-based disability, this means that the current autistic sample had relatively low support needs during engagement with the study. For people with higher support needs in relation to reading, the inclusion of texts being read aloud may pose different benefits and drawbacks. In particular, less experienced readers in this study tended to find the audio helpful for difficult texts, indicating a benefit where readers might broadly struggle with reading. Therefore, there is a need for future research to explore the reading experiences of autistic people from a wider range of backgrounds. In particular, there is a need to understand how autistic people who communicate through alternative, non-verbal means of communication would benefit personally from reading serious literature and in subsequently reflecting with other readers. This is because autistic people who use augmented and alternative communication methods are currently underrepresented in research and are likely to have different experiences of developing mutuality in everyday socio-communication.

Furthermore, readers in the current study read alone, meaning that further research would be needed to understand how autistic and non-autistic readers may comparatively apply their experiences in broader situations. Therefore, conclusions around autistic and non-autistic social processing differences are limited to the reading experiences outlined in this study. Future research would then benefit from longitudinal explorations of autistic and non-autistic reading experiences and any resultant real-world changes. Current findings that pre-recorded readings did not elicit the benefits of live reading together with previous findings that autistic people are uncomfortable with in-person live readings (Chapple et al., 2021b) indicate that further exploration is required before designing reading aloud groups for use with autistic and non-autistic readers. Future research should then explore how a live, distanced online design could overcome concerns and whether such a design would facilitate the benefits of live reading aloud groups.

## 5. Conclusion

In conclusion, the findings presented in this study challenge long-held social deficit views of autism (for example: Baron-Cohen, 1997, 2008; Happé, 1999), by showing that both the autistic and non-autistic readers were able to engage with the social breadth and depth of fictional social realities and meaningfully share their responses. Additionally, current findings further support earlier research in showing that autistic people may be more literary readers who, when moved, were especially capable of working with the experience of uncertainty and not-knowing, where non-autistic readers had readier recourse to assured competence (Chapple et al., 2022). The serious literature in the current study was able to encourage autistic readers to start to see the value in struggling and holding onto the intangible or not easily nameable. In this study, the social processing differences between autistic and non-autistic participants came at the storing and recall stage of the reading experience. Specifically, autistic readers better held onto the complex detail and resonance of the inter-personal experience of the literature, while non-autistic readers seemed more likely to extract core ideas and meanings for generalization across situations by means of data reduction. Further research is needed to understand the specific advantages and disadvantages that may then result in drawing on the reading experience for real-world social processing. Together with previous research looking at shared reading reflections between autistic and non-autistic readers (Chapple et al., 2021a), it appears that inter-neurotype discussions around the literature are needed to promote mutuality for double empathy (Milton, 2012). In considering how reading aloud designs could enhance these shared reading methodologies aiming to promote double empathy, the current study highlights that further exploration is needed. Specifically, the current study demonstrates that pre-recorded readings did not bring about the benefits of live shared reading (Longden et al., 2015). Overall, the findings here further highlight the ability of serious literature in particular to challenge dominant thinking about autism, moving toward more inclusive understandings of social processing differences.

## Data availability statement

The datasets presented in this article are not readily available because the data used for the current study contained sensitive information that could be used to identify the participants. Quotes have been presented in the manuscript that do not include any of this sensitive information, but the larger data set is not suitable for public availability. Requests to access the datasets should be directed to MC, melissa.chapple@liverpool.ac.uk.

## Ethics statement

The studies involving human participants were reviewed and approved by the University of Liverpool Ethics Department. The patients/participants provided their written informed consent to participate in this study.

## Author contributions

MC, PD, JB, and RC: conceptualization, methodology, validation, formal analysis, and writing—review and editing. MC: funding acquisition, data curation, project administration, and writing—original draft. PD, JB, and RC: supervision. All authors contributed to the article and approved the submitted version.

## Footnotes

1 This article uses identity-first language (i.e., autistic people) due to existing literature which suggest that identity-first language best meets the preferences of the broader autistic community and prevents understandings of autism that Other autistic people (Kenny et al., 2016; Botha et al., 2021; Bottema-Beutel et al., 2021; Monk et al., 2022).

2 Experienced reader is used to refer to a literary specialist with extensive experience in reading texts aloud.

3 This article was taken from Mint in 2018 and has since been removed from the website. The extract is attached as Supplementary Appendix 1.

## References

[B1] American Psychiatric Association (2013). *Neurodevelopmental disorders. In Diagnostic and statistical manual of mental disorders*, 5th Edn. Virginia, US: American Psychiatric Association.

[B2] Ammons R. B. Ammons C. H. (1962). The quick test (QT): Provisional manual. *Psychol. Rep.* 11 111–118. 10.1177/003329416201100106

[B3] Armstrong R. M. Paynter J. Westerveld M. F. (2019). Fiction or non-fiction: Parent-reported book preferences of their preschoolers with autism spectrum disorder. *Autism Dev. Lang. Impairments* 4:2396941519896736.

[B4] Arnaud S. (2022). Self-consciousness in autism: A third-person perspective on the self. *Mind Lang.* 37 356–372. 10.1111/mila.12356

[B5] Barnes J. L. (2012). Fiction, imagination, and social cognition: Insights from autism. *Poetics* 40 299–316.

[B6] Barnes J. L. (2018). Imaginary engagement, real-world effects: Fiction, emotion, and social cognition. *Rev. Gener. Psychol.* 22 125–134. 10.1017/S0963180116000578 27852344

[B7] Baron-Cohen S. (1997). *Mindblindness: An essay on autism and theory of mind.* Cambridge, MA: MIT Press.

[B8] Baron-Cohen S. (2002). The extreme male brain theory of autism. *Trends Cogn. Sci.* 6 248–254. 10.1016/S1364-6613(02)01904-6 12039606

[B9] Baron-Cohen S. (2008). *Autism and Asperger syndrome.* New York, NY: Oxford University Press.

[B10] Baron-Cohen S. (2009). The empathising-systemising theory of autism: Implications for education. *Tizard Learn. Disabil. Rev.* 14 4–13. 10.1108/13595474200900022

[B11] Baron-Cohen S. Wheelwright S. Skinner R. Martin J. Clubley E. (2001). The autism-spectrum quotient (AQ): Evidence from Asperger syndrome/high-functioning autism, males and females, scientists and mathematicians. *J. Autism Dev. Disord.* 31 5–17. 10.1023/A:1005653411471 11439754

[B12] Billington J. Davis P. Farrington G. Green K. Magee F. Steenberg M. (2019). “Qualitative methods: Developing innovative qualitative approaches in research on reading and health,” in *Reading and mental health*, ed. Billington J. (London, UK: Palgrave Macmillan), 191–240.

[B13] Bodner K. E. Engelhardt C. R. Minshew N. J. Williams D. L. (2015). Making inferences: Comprehension of physical causality, intentionality, and emotions in discourse by high-functioning older children, adolescents, and adults with autism. *J. Autism Dev. Disord.* 45 2721–2733. 10.1007/s10803-015-2436-3 25821925 PMC4555006

[B14] Botha M. (2021). Academic, activist, or advocate? angry, entangled, and emerging: A critical reflection on autism knowledge production. *Front. Psychol.* 12:727542. 10.3389/fpsyg.2021.727542 34650484 PMC8506216

[B15] Botha M. Hanlon J. Williams G. L. (2021). Does language matter? Identity-first versus person-first language use in autism research: A response to Vivanti. *J. Autism Dev. Disord.* 53 870–878. 10.1007/s10803-020-04858-w 33474662 PMC7817071

[B16] Bottema-Beutel K. Kapp S. K. Lester J. N. Sasson N. J. Hand B. N. (2021). Avoiding ableist language: Suggestions for autism researchers. *Autism Adulthood* 3 18–29. 10.1089/aut.2020.0014 36601265 PMC8992888

[B17] Burrows C. A. Usher L. V. Mundy P. C. Henderson H. A. (2017). The salience of the self: Self-referential processing and internalizing problems in children and adolescents with autism spectrum disorder. *Autism Res.* 10 949–960. 10.1002/aur.1727 27868365 PMC5438783

[B18] Burton H. (2013). *Exploring autism: A conversation with Uta Frith.* Brussels: Open Agenda Publishing.

[B19] Chapple M. Davis P. Billington J. Myrick J. A. Ruddock C. Corcoran R. (2021a). Overcoming the double empathy problem within pairs of autistic and non-autistic adults through the contemplation of serious literature. *Front. Psychol.* 12:708375. 10.3389/fpsyg.2021.708375 34385964 PMC8354525

[B20] Chapple M. Williams S. Billington J. Davis P. Corcoran R. (2021b). An analysis of the reading habits of autistic adults compared to neurotypical adults and implications for future interventions. *Res. Dev. Disabil.* 115:104003. 10.1016/j.ridd.2021.104003 34116300

[B21] Chapple M. Davis P. Billington J. Williams S. Corcoran R. (2022). Challenging empathic deficit models of autism through responses to serious literature. *Front. Psychol.* 13:828603. 10.3389/fpsyg.2022.828603 35222208 PMC8867167

[B22] Chown N. (2014). More on the ontological status of autism and double empathy. *Disabil. Soc.* 29 1672–1676. 10.1080/09687599.2014.949625

[B23] Clarke V. Braun V. (2014). “Thematic analysis,” in *Encyclopedia of critical psychology*, ed. Teo T. (New York, NY: Springer), 1947–1952.

[B24] Crompton C. J. Ropar D. Evans-Williams C. V. Flynn E. G. Fletcher-Watson S. (2020b). Autistic peer-to-peer information transfer is highly effective. *Autism* 24 1704–1712. 10.1177/1362361320919286 32431157 PMC7545656

[B25] Crompton C. J. Hallett S. Ropar D. Flynn E. Fletcher-Watson S. (2020a). ‘I never realised everybody felt as happy as I do when I am around autistic people’: A thematic analysis of autistic adults’ relationships with autistic and neurotypical friends and family. *Autism* 24 1438–1448. 10.1177/1362361320908976 32148068 PMC7376620

[B26] Crompton C. J. Sharp M. Axbey H. Fletcher-Watson S. Flynn E. G. Ropar D. (2020c). Neurotype-matching, but not being autistic, influences self and observer ratings of interpersonal rapport. *Front. Psychol.* 11:586171. 10.3389/fpsyg.2020.586171 33192918 PMC7645034

[B27] Davidson M. M. Ellis Weismer S. (2018). A preliminary investigation of parent-reported fiction versus non-fiction book preferences of school-age children with autism spectrum disorder. *Autism Dev. Lang. Impairments* 3:2396941518806109. 10.1177/2396941518806109 30733999 PMC6363357

[B28] Davis P. (2020). *Reading for life.* New York, NY: Oxford University Press.

[B29] Davis P. Magee F. (2020). *Reading.* Bingley, UK: Emerald Publishing.

[B30] DeBrabander K. M. Morrison K. E. Jones D. R. Faso D. J. Chmielewski M. Sasson N. J. (2019). Do first impressions of autistic adults differ between autistic and non-autistic observers? *Autism Adulthood* 1 250–257. 10.1089/aut.2019.0018 36601322 PMC8992824

[B31] Dickens C. (2012). *Great expectations.* London: UK: Penguin Books.

[B32] Dinishak J. Akhtar N. (2013). A critical examination of mindblindness as a metaphor for autism. *Child Dev. Perspect.* 7 110–114. 10.1111/cdep.12026

[B33] Djikic M. Oatley K. Moldoveanu M. C. (2013). Opening the closed mind: The effect of exposure to literature on the need for closure. *Creat. Res. J.* 25 149–154. 10.1080/10400419.2013.783735

[B34] Doyle A. C. (2012). *The memoirs of Sherlock Holmes.* New York, NY: Random House Group.

[B35] Edey R. Cook J. Brewer R. Johnson M. H. Bird G. Press C. (2016). Interaction takes two: Typical adults exhibit mind-blindness towards those with autism spectrum disorder. *J. Abnorm. Psychol.* 125:879. 10.1037/abn0000199 27583766

[B36] Farrington G. Davis P. Billington J. (2019). “The Victorian novel: Laying the foundations for ‘bibliotherapy’,” in *Reading and mental health*, ed. Billington J. (London, UK: Palgrave Macmillan.), 47–71.

[B37] Fletcher-Watson S. Bird G. (2020). Autism and empathy: What are the real links? *Autism* 24 3–6. 10.1177/1362361319883506 31674189

[B38] Fletcher-Watson S. Happé F. (2019). *Autism: A new introduction to psychological theory and current debate.* London, UK: Routledge.

[B39] Gaigg S. B. (2012). The interplay between emotion and cognition in autism spectrum disorder: Implications for developmental theory. *Front. Integr. Neurosci.* 6:113. 10.3389/fnint.2012.00113 23316143 PMC3540960

[B40] Green S. Davis C. Karshmer E. Marsh P. Straight B. (2005). Living stigma: The impact of labeling, stereotyping, separation, status loss, and discrimination in the lives of individuals with disabilities and their families. *Sociol. Inq.* 75 197–215. 10.1111/j.1475-682X.2005.00119.x

[B41] Happé F. (1999). Autism: Cognitive deficit or cognitive style? *Trends Cogn. Sci.* 3 216–222. 10354574 10.1016/s1364-6613(99)01318-2

[B42] Harmsen I. E. (2019). Empathy in autism spectrum disorder. *J. Autism Dev. Disord.* 49 3939–3955. 10.1007/s10803-019-04087-w 31147901

[B43] Harris J. (2010). “Faith and Hope go shopping,” in *A little aloud*, ed. Macmillan A. (London, UK: Random House Group), 409–422.

[B44] Heasman B. Gillespie A. (2018). Perspective-taking is two-sided: Misunderstandings between people with Asperger’s syndrome and their family members. *Autism* 22 740–750. 10.1177/1362361317708287 28683569 PMC6055325

[B45] Heasman B. Gillespie A. (2019). Neurodivergent intersubjectivity: Distinctive features of how autistic people create shared understanding. *Autism* 23 910–921. 10.1177/1362361318785172 30073872 PMC6512057

[B46] Hill E. L. (2004). Executive dysfunction in autism. *Trends Cogn. Sci.* 8 26–32. 10.1016/j.tics.2003.11.003 14697400

[B47] Honeyman G. (2017). *Eleanor Oliphant is completely fine.* London: UK: Harper Collins.

[B48] Hume R. Burgess H. (2021). “I’m human after all”: Autism, trauma, and affective empathy. *Autism Adulthood* 3 221–229. 10.1089/aut.2020.0013 36605372 PMC8992898

[B49] Ida D. (2020). “Multiplicity and neurodiversity – exploring potential in Deleuzoguattarian social theory for furthering a paradigm shift,” in *The neurodiversity reader*, eds Milton D. Ridout S. Martin N. Mills R. Murray D. (West Sussex, UK: Pavilion), 78–94.

[B50] Kaszynska P. (2015). Capturing the vanishing point: Subjective experiences and cultural value. *Cult. Trends* 24 256–266. 10.1080/09548963.2015.1066077

[B51] Kenny L. Hattersley C. Molins B. Buckley C. Povey C. Pellicano E. (2016). Which terms should be used to describe autism? Perspectives from the UK autism community. *Autism* 20 442–462. 10.1177/1362361315588200 26134030

[B52] Kinderman P. Read J. Moncrieff J. Bentall R. P. (2013). Drop the language of disorder. *Evid. Based Ment. Health* 16 2–3. 23002095 10.1136/eb-2012-100987

[B53] Koopman E. M. Hakemulder F. (2015). Effects of literature on empathy and self-reflection: A theoretical-empirical framework. *J. Lit. Theory* 9 79–111. 10.1515/jlt-2015-0005

[B54] Lesser M. Murray D. (2020). “Mind as a dynamical system – implications for autism,” in *The neurodiversity reader*, eds Milton D. Ridout S. Martin N. Mills R. Murray D. (West Sussex, UK: Pavilion), 7–17.

[B55] Limburg J. (2021). *Letters to my weird sisters.* London: UK: Atlantic Books.

[B56] Lombardo M. V. Baron-Cohen S. (2011). The role of the self in mindblindness in autism. *Conscious. Cogn.* 20 130–140. 10.1016/j.concog.2010.09.006 20932779

[B57] Longden E. Davis P. Billington J. Lampropoulou S. Farrington G. Magee F. (2015). Shared reading: Assessing the intrinsic value of a literature-based health intervention. *Med. Hum.* 41 113–120. 10.1136/medhum-2015-010704 26070845

[B58] Macmillan A. (2010). *A little aloud.* New York, NY: Random House Group.

[B59] Mar R. A. Oatley K. (2008). The function of fiction is the abstraction and simulation of social experience. *Perspect. Psychol. Sci.* 3 173–192. 10.1111/j.1745-6924.2008.00073.x 26158934

[B60] McCartney P. (2021). “Tell me who he is,” in *The Lyrics*, ed. Muldoon P. (New York, NY: Penguin Random House), 700–701.

[B61] Milton D. Ridout S. Martin N. Mills R. Murray D. (eds) (2020). *The neurodiversity reader.* West Sussex, UK: Pavilion.

[B62] Milton D. E. (2012). On the ontological status of autism: The ‘double empathy problem’. *Disabil. Soc.* 27 883–887. 10.1080/09687599.2012.710008

[B63] Milton D. E. M. Heasman B. Sheppard E. (2018). “Double Empathy,” in *Encyclopedia of Autism Spectrum Disorders*, ed. Volkmar F. (New York, NY: Springer), 10.1007/978-1-4614-6435-8_102273-1

[B64] Monk R. Whitehouse A. J. Waddington H. (2022). The use of language in autism research. *Trends Neurosci.* 45 791–793. 10.1016/j.tins.2022.08.009 36184384

[B65] Morrison K. E. DeBrabander K. M. Jones D. R. Faso D. J. Ackerman R. A. Sasson N. J. (2020). Outcomes of real-world social interaction for autistic adults paired with autistic compared to typically developing partners. *Autism* 24 1067–1080. 10.1177/1362361319892701 31823656

[B66] Mumper M. L. Gerrig R. J. (2019). How does leisure reading affect social cognitive abilities? *Poetics Today* 40 453–473. 10.1215/03335372-7558080

[B67] Murray D. Lesser M. Lawson W. (2005). Attention, monotropism and the diagnostic criteria for autism. *Autism* 9 139–156. 10.1177/1362361305051398 15857859

[B68] Murray F. (2020). “Neurodiversity is for everyone,” in *The neurodiversity reader*, eds Milton D. Ridout S. Martin N. Mills R. Murray D. (West Sussex, UK: Pavilion), 105–109.

[B69] O’Sullivan N. Davis P. Billington J. Gonzalez-Diaz V. Corcoran R. (2015). “Shall I compare thee”: The neural basis of literary awareness, and its benefits to cognition. *Cortex* 73 144–157. 10.1016/j.cortex.2015.08.014 26409018

[B70] Packham C. (2017). *Freedom to be honest.* London: Your Autism Magazine.

[B71] Pearson A. Rose K. (2021). A conceptual analysis of autistic masking: Understanding the narrative of stigma and the illusion of choice. *Autism Adulthood* 3 52–60. 10.1089/aut.2020.0043 36601266 PMC8992880

[B72] Richards I. A. (2017). *Practical criticism: A study of literary judgement.* London: Routledge.

[B73] Rigby S. N. Stoesz B. M. Jakobson L. S. (2018). Empathy and face processing in adults with and without autism spectrum disorder. *Autism Res.* 11 942–955. 10.1002/aur.1948 29637718

[B74] Rumball F. Brook L. Happé F. Karl A. (2021). Heightened risk of posttraumatic stress disorder in adults with autism spectrum disorder: The role of cumulative trauma and memory deficits. *Res. Dev. Disabil.* 110:103848. 10.1016/j.ridd.2020.103848 33454451

[B75] Schriber R. A. Robins R. W. Solomon M. (2014). Personality and self-insight in individuals with autism spectrum disorder. *J. Person. Soc. Psychol.* 106:112. 10.1037/a0034950 24377361 PMC4122539

[B76] Sheppard E. Pillai D. Wong G. T. L. Ropar D. Mitchell P. (2016). How easy is it to read the minds of people with autism spectrum disorder? *J. Autism Dev. Disord.* 46 1247–1254. 10.1007/s10803-015-2662-8 26603886

[B77] Smith A. (2009). The empathy imbalance hypothesis of autism: A theoretical approach to cognitive and emotional empathy in autistic development. *Psychol. Record* 59 489–510.

[B78] Smith J. (2018). How selfish is your search for happiness? *Psychologist* 31 28–32.

[B79] Song Y. Nie T. Shi W. Zhao X. Yang Y. (2019). Empathy impairment in individuals with autism spectrum conditions from a multidimensional perspective: A meta-analysis. *Front. Psychol.* 10:1902. 10.3389/fpsyg.2019.01902 31649570 PMC6794557

[B80] Strick M. Van Soolingen J. (2018). Against the odds: Human values arising in unfavourable circumstances elicit the feeling of being moved. *Cogn. Emot.* 32 1231–1246. 10.1080/02699931.2017.1395729 29083264

[B81] Waltz M. (2013). *Autism a social and medical history.* Hampshire, UK: Palgrave Macmillan.

[B82] Waytz A. Hershfield H. E. Tamir D. I. (2015). Mental simulation and meaning in life. *J. Person. Soc. Psychol.* 108:336. 10.1037/a0038322 PMC4480924 25603379

[B83] Zunshine L. (2011). Style brings in mental states. *Style* 45 349–356. 10.5325/style.45.2.349

