# Supplementary material for: Exploring the different cognitive, emotional and imaginative experiences of autistic and non-autistic adult readers when contemplating serious literature as compared to non-fiction
Source: Front Psychol. 2023 May 4;14:1001268. doi: 10.3389/fpsyg.2023.1001268 (PMC10192853; doi:10.3389/fpsyg.2023.1001268)
Supplement: Supplementary file 1 [file Data_Sheet_1.docx]

**Appendix 1**

**Extract from ‘Expert Interview with Gretchen Rubin on Finding Happiness for Mint’**

Despite the fact that she's spent years studying the concept of happiness and how we can get happy, bestselling author and blogger Gretchen Rubin refuses to ascribe an exact definition to the word.

It's not that she doesn't understand the usefulness of precise definitions - she once spent an entire semester in law school discussing the meaning of a "contract" - and she cites one positive psychology study that identified 15 different academic definitions of happiness. But for her, spending a lot of energy exploring the distinctions among "positive affect," "subjective well-being," "hedonic tone," and myriad of other terms didn't seem necessary.

Instead, she followed the hallowed tradition set by Supreme Court Justice Potter Stewart, who defined obscenity by saying, "I know it when I see it," or Louis Armstrong, who said, "If you have to ask what jazz is, you'll never know."

"Even people who can't agree on what it means to be 'happy' can agree that most people can be 'happier,' according to their own particular definition," she said. "I know when I feel happier. That was good enough for my purposes."

Gretchen, whose book The Happiness Project has sold more than 1.5 million copies in North America alone, recently checked in with us to discuss (what else?) happiness. More specifically, ideas for how we can get happy, the biggest roadblocks to our own happiness and reflections on how happiness and money relate to each other. Read on for her take:

**Tell us about The Happiness Project...what is it? Why did you start it?**

One April day, on a morning just like every other morning, I had a sudden realization: I was in danger of wasting my life. As I stared out the rain-spattered window of a city bus, I saw that the years were slipping by. "What do I want from life, anyway?" I asked myself. "Well...I want to be happy." But I had never thought about what made me happy, or how I might be happier.

I had much to be happy about. I had a great husband and two delightful young daughters; I was a writer, after starting out as a lawyer; I was living in my favourite city, New York; I had close relationships with my parents, sister and in-laws; I had friends; I had my health; I didn't even have to colour my hair. But too often I sniped at my husband or the cable guy. I felt dejected after even a minor professional setback. I drifted out of touch with old friends, I lost my temper easily.

I wasn't depressed, and I wasn't having a midlife crisis, but I was suffering an adulthood malaise - a recurrent sense of discontent, and almost a feeling of disbelief.

But though at times I felt dissatisfied, that something was missing, I also never forgot how fortunate I was. I had everything I could possibly want - yet I was failing to appreciate it. I didn't want to keep taking these days for granted.

"I've got to tackle this," I reflected. "As soon as I have some free time, I should start a happiness project." But I never had any free time. When life was taking its ordinary course, it was hard to remember what really mattered; if I wanted a happiness project, I'd have to make the time.

I grasped two things: I wasn't as happy as I could be, and my life wasn't going to change unless I made it change. In that single moment, with that realization, I decided to dedicate a year to trying to be happier.

Every month, for a year, I tackled one area that would boost my happiness, such as marriage, parenthood, energy, mindfulness, leisure and work.

I gained so much from doing this happiness project that I did a second one, to focus on being happier at home - which I wrote about in a book called, appropriately enough, Happier at Home. I realized that for just about everyone, home had a special role to play in a happy life. I dug deep into areas such as div, possessions, family and neighbourhood.

**Why do you think we struggle so much with finding and understanding our own happiness?**

Before I started my happiness project, I never spent any time thinking about happiness, or what I could do to be happier - and I think that's very common. It takes a lot of reflection, and sometimes painful self-knowledge, to figure out our own interests, values and nature.

**What are some of the biggest complaints, concerns or questions your readers come to you with about happiness?**

I noticed that an issue that comes up over and over is habit-formation. When people talk about a big happiness challenge that they struggle with, or a big boost they've managed to make in their happiness, very often they talk about their habits.

That's why my next book, Better Than Before, is about how we make and break habits. Whether it's getting more sleep, exercising regularly, turning off a cellphone, finishing a Ph.D. thesis, or meditating, changing a habit allow us to change our lives.

**What are the steps the average person needs to take in the journey to happiness?**

First, identify your aims. Ask yourself:

- What makes you feel good? What gives you joy, energy, fun?
- What makes you feel bad? What brings you anger, guilt, boredom, dread?
- What makes you feel right? What values do you want your life to reflect?
- How can you build an atmosphere of growth - where you learn, explore, build, teach, help?

Next, make resolutions to build habits that are concrete and manageable. "Play with my dog each morning" is more effective than "Get more fun out of life."

Track your resolutions by finding a way to hold yourself accountable.

**Where do you find the fuel for your happiness - are there books you read? Activities you participate in? People who re-energize you?**

All these things! I love to read, and I spent a huge amount of time reading any book that touches on my subjects - happiness, and now habits. Whenever I come across an idea that resonates with me, I test it in my own life. And I get a huge amount of energy and ideas from talking to the people around me.

**What have you learned about the relationship between happiness and money?**

The relationship between money and happiness was one of the most interesting, most complicated and most sensitive questions in my study of happiness.

We often see the argument, "Money can't buy happiness," but it certainly seems that, whatever any economist or social scientist might claim, people appear fairly well convinced that money matters to their happiness.

So, am I arguing that "Money can buy happiness?" The answer: No. That's clear. Money, alone, can't buy happiness.

But can money help buy happiness? The answer: Yes, used wisely, it can. Whether rich or poor, people make choices about how they spend money, and those choices can boost happiness or undermine happiness. You might buy cocaine, or you might buy a dog. You might splurge on a big-screen TV, or you might splurge on a new bike.

Money affects people in different ways. No statistical average can say how a particular individual would be affected by money - depending on that individual's circumstances and temperament. You might live in an expensive big city, or in the country. You might have aging parents and several young children, or you might be single. You might love to travel and ride horses, or you might love to watch movies at home. What matters are our choices and habits.

**How do you think the age of social media and constant exposure to other people's personal lives has affected our happiness?**

This is a subject of tremendous study right now. It's a fascinating question.

Most people tend to emphasize the downsides of social media - for instance, that people feel bad when they compare their lives to the shiny picture presented of other people's lives.

From my own experience, though, I'd say that the good outweighs the bad. One of the most important elements of a happy life - probably the most important element - is strong relationships with other people.
